# Novel Insights into the Ontogeny of Nestmate Recognition in *Polistes* Social Wasps

**DOI:** 10.1371/journal.pone.0097024

**Published:** 2014-05-07

**Authors:** Lisa Signorotti, Federico Cappa, Patrizia d’Ettorre, Rita Cervo

**Affiliations:** 1 Laboratory of Experimental and Comparative Ethology (LEEC), University of Paris 13- Sorbonne Paris Cité, Villetaneuse, France; 2 Dipartimento di Biologia, Università degli Studi di Firenze, Sesto Fiorentino, Italy; Universidade de São Paulo, Faculdade de Filosofia Ciências e Letras de Ribeirão Preto, Brazil

## Abstract

The importance of early experience in animals’ life is unquestionable, and imprinting-like phenomena may shape important aspects of behaviour. Early learning typically occurs during a sensitive period, which restricts crucial processes of information storage to a specific developmental phase. The characteristics of the sensitive period have been largely investigated in vertebrates, because of their complexity and plasticity, both in behaviour and neurophysiology, but early learning occurs also in invertebrates. In social insects, early learning appears to influence important social behaviours such as nestmate recognition. Yet, the mechanisms underlying recognition systems are not fully understood. It is currently believed that *Polistes* social wasps are able to discriminate nestmates from non-nestmates following the perception of olfactory cues present on the paper of their nest, which are learned during a strict sensitive period, immediately after emergence. Here, through differential odour experience experiments, we show that workers of *Polistes dominula* develop correct nestmate recognition abilities soon after emergence even in absence of what have been so far considered the necessary cues (the chemicals spread on nest paper). *P. dominula* workers were exposed for the first four days of adult life to paper fragments from their nest, or from a foreign conspecific nest or to a neutral condition. Wasps were then transferred to their original nests where recognition abilities were tested. Our results show that wasps do not alter their recognition ability if exposed only to nest material, or in absence of nest material, during the early phase of adult life. It thus appears that the nest paper is not used as a source of recognition cues to be learned in a specific time window, although we discuss possible alternative explanations. Our study provides a novel perspective for the study of the ontogeny of nestmate recognition in *Polistes* wasps and in other social insects.

## Introduction

Early experiences in life can have significant consequences on the behaviour of animals and on their survival. Since the pioneer work of Konrad Lorenz [1] “critical” or sensitive periods in neural, cognitive and behavioural development have been largely investigated, focusing on peculiar forms of learning such as imprinting or imprinting-like phenomena [2]. The restriction of learning to a sensitive temporal window during ontogeny allows the acquisition of biologically relevant information while reducing the risk of evaluation errors. The most suitable learning period, in general, corresponds to an early phase of the individual’s life. For example, in ducks and chickens auditory and visual stimuli that identify the parents are learned within a few days from hatching [3], [4], being the parents the first individuals met in natural conditions. These stimuli are later used to recognize and follow the parents but also to recognize and avoid other adults as well as heterospecifics that could be potential predators [5].

The characteristics of the sensitive period have typically been investigated in birds and mammals because of their complexity and plasticity, both in behaviour and in the neural machinery at the basis [6]. Nonetheless, the existence of sensitive windows for learning has also been demonstrated in invertebrates, including social insects, with a critical role in shaping recognition abilities and social interactions [7].

In social insects, the ability to recognize nestmates (individuals belonging to the own colony) plays a critical role in the maintenance of cooperative behaviour [8]. Nestmate recognition is mediated by chemical cues (i.e. a blend of cuticular hydrocarbons, CHCs, covering the body surface of each individual) that are qualitatively similar in a given species but can vary in their relative amounts among colonies of the same species [9], [10], [11], [12]. According to the phenotype matching model, social insects discriminate among nestmates and alien individuals by comparing the chemical cues perceived on the body surface of the encountered individual (CHCs profile) with a neural “template” (referent colony odour previously learned) [13], [14].

Several studies have investigated the role, timing and form of learning underlying the ontogeny of nestmate recognition in social insects [15], [16], [17], [18]. *Polistes* paper wasps have been used as a traditional model in these studies, and the acknowledged idea is that each wasp learns the olfactory recognition cues from the paper of their natal nest during a strict sensitive window, namely the first few hours after emergence [19], [20]. The nest material conveys the same chemicals of the colony inhabitants, providing information about colony membership [21], [22], [23], [24], [25], and it is therefore considered to be the primary source of cues for the acquisition of the referent template [26], [27]. Although it is generally believed that the nestmate recognition mechanism is shared within the *Polistes* genus [28], [29], [30], to date there is no evidence that in *Polistes dominula*, a model species for nestmate recognition in the genus [11], such mechanism follows the same rules. The eusocial lifestyle likely facilitates the development of common features in the mechanisms at the basis, but different factors (e.g., colony kin structure, environmental pressure,) could shape alternative patterns of recognition in different species [31]. Moreover, in the context of ontogeny of nestmate recognition, only American species of the subgenus *Aphanilopterus* [32] have been experimentally tested so far [28], [29], [30], while *P. dominula* belongs to *Polistes sensu stricto* [32].

Here, for the first time, we investigated whether in *P. dominula* wasps the early olfactory experience through contact with nest material is a fundamental prerequisite for the development of correct recognition abilities. We experimentally exposed pre-eclosing workers to their natal nest material, to foreign nest material, or to neutral filter paper, during four days in absence of nestmates. Afterwards, the experimental wasps were transferred onto their original nests and bioassays were performed to assess their recognition abilities towards nestmate, alien or familiar (i.e. coming from the foreign nest that provided the material) lure wasps. If the *Polistes* recognition model applies to all the *Polistes* species, we predict that: 1) wasps in contact with their natal nest material during the early phase of their adult life should develop correct nestmate recognition abilities (i.e. non aggressive towards nestmates whereas aggressive towards alien individuals); 2) wasps in contact with foreign nest material in the early phase of their adult life should show incorrect nestmate recognition (i.e. less aggressive towards individuals belonging to the foreign nest (“familiar”) with respect to actual nestmates and completely unfamiliar alien wasps); 3) wasps not exposed to nest material during the early phase of their adult life should be unable to form a referent template and thus unable to perform a correct nestmate discrimination. Our results challenge this model.

## Materials and Methods

### Ethic Statement

The collection of colonies and the performed behavioural experiments comply with the current laws in Italy. No specific permits are required for collection of wasps, and the species used in the experiments is not endangered or protected in Italy.

### Study Species

*Polistes dominula* (Christ) is the most common species of the genus *Polistes* among Old World species, with a native range from Europe to China [33], [34]. Nevertheless, recently, by accidental introductions, it invaded the New World, both in North and South America, expanding its original range [34], [35]. The colony cycle starts in springtime when the inseminated females (foundresses) emerge from hibernacula and found a new nest. Nests can be founded either by a single or by a group of foundresses (associative foundation). During the founding phase, co-foundresses coexist and establish a linear dominancy hierarchy [36], [37] that mirrors the division of labour and the reproductive skew in the colony: the dominant female remains on the nest, dominates the associates and lays the majority of eggs [38], while the subordinate females behave as worker force and renounce to their direct fitness [36], [37]. The first generation of workers starts to emerge at the end of May, whereas males and reproductive females emerge in late summer and leave the nests to mate. Colonies are proterandric in the production of sexuals, therefore females eclosing before the emergence of males are considered workers, even though workers emerge throughout the colony cycle. After the mating period (mid- to late Autumn), males die and inseminated females (future foundresses) entre in diapauses until the next spring season when they will start a new colony cycle [33].

### Colonies Collection and Laboratory Rearing

Associative foundations of *P. dominula* (n = 38) were collected in late June 2013 in different localities of Tuscany (Italy). We collected colonies in which the first generation of workers had already eclosed. Nests had approximately 80 cells and contained immature brood (i.e. eggs, larvae and pupae). Colonies were transferred in glass boxes (15×15×15 cm) and provided with sugar, larvae of *Tenebrio molitor* and water *ad libitum.* Boxes were kept in the laboratory under natural photoperiod at ∼25C° for 2 weeks. Foundresses and workers, found on the nests at collection, were marked on the wings with acrylic colours (Testor Enamel) to distinguish them from newly emerging workers. After four days, when marked adults were at least 3 days old, a time window essential to allow the development of a complete cuticular chemical profile in this species [39], five marked workers were removed from each nest and killed by freezing to be used later as lure wasps in recognition bioassays.

### Selection of Experimental Pre-eclosing Wasps

Paper wasps at the end of their pupal development cut the cell cups with their mandibles immediately before emergence. Soon after eclosion, newly emerged individuals get in contact with their natal comb and nestmates, having the opportunity to learn chemical cues useful for the development of their nestmate recognition abilities. The main purpose of our study was to experimentally manipulate the first olfactory experience of adult individuals during the phase that is considered critical for learning, namely the first hours after emergence [29]. Therefore, we developed a method to remove workers from their natal nest at the end of their pupal stage (just before eclosion), to ensure that they were not exposed to their colony odour in the early phase of their adult life. To evaluate the correct timing to select emerging individuals from the natal combs, we partially removed the cell cups before wasps’ emergence with clean forceps and we observed the colour and the movements of pre-eclosing workers. We selected as experimental individuals, pre-eclosing workers with both bright yellow/black colours and moving heads and antennae. After cell uncapping, wasps were left in their own natal cell and monitored for emergence every 10 min for up to four hours and then the day after. All the 25 individuals monitored for this preliminary observation emerged within 24 hours from cell uncapping. The result confirmed that our criteria (i.e. colour and movements of head and antennae), used to select pre-emerging wasps, were accurate allowing us to collect individuals that will emerge within a day.

### Experimental Design

We monitored each nest for pre-eclosing wasps from 1^st^ to 10^th^ July by partially removing the cell cups of pupae, as explained above. Wasps emerging at times when nests were not monitored were not used in this study. Pre-eclosing workers, that met the selected criteria mentioned above, were gently removed from their cells with soft tweezers and transferred individually into small plastic Petri dishes (diameter 2×1.5 cm). Experimental workers were divided into three groups: 1) 40 wasps (“N” = Neutral) were transferred to individual Petri dishes containing a piece of filter paper (2.5 cm^2^); 2) 40 wasps (“C” = Control) were transferred to Petri dishes containing own nest material (corresponding to about three empty nest cells) and a piece of filter paper (2×1 cm); 3) 40 wasps (“F” = Familiarized) were transferred to Petri dishes containing nest material from a foreign unrelated nest and a piece of filter paper (2×1 cm). The filter paper was previously washed with pentane for 15 minutes in order to remove any contaminations. Each Petri dish was provided with a hole for air entrance and with a small candy as food for the newly-eclosed wasp. Wasps of the three groups experienced a different odour exposure during the early hours of their adult life: “N” workers were exposed to no odours; conversely, “C” and “F” workers were exposed to the odour of their natal nest and of a foreign nest respectively. To guarantee that the sensitive phase for learning (few hours after emergence) reported for other *Polistes* species [19], [20] was included in our experimental temporal window, wasps were left in Petri dishes for four days. Each wasp was then individually marked, transferred back to their natal nest and tested in recognition bioassays the following day, to allow acclimatization.

### Recognition Bioassays

Before starting the bioassays, we removed all the resident wasps from each nest except the experimental individual. Each experimental wasp was left undisturbed on its nest for at least 15 min. Experimental wasps that did not remain on their nests within 20 minutes from the removal of nestmates (about 37%) were not used for bioassays. A total of seventy-five experimental wasps were tested: 24 “C”, 25 “N” and 26 “F” workers. To evaluate the recognition abilities of experimental individuals, we presented each wasp with three different kinds of lures, i.e. the body of dead wasps freshly killed by freezing, and we recorded their behavioural responses. For the “N” and “C” experimental wasps one lure was represented by a nestmate while the other two were alien wasps from foreign colonies (designated as alien 1 and alien 2 in the results section) collected several kilometres apart to avoid any relatedness with tested wasps. For the “F” group, one lure was represented by a nestmate, one by a wasp belonging to the colony that provided nest fragments for the exposure phase (familiar), and the third one by an alien, completely unfamiliar wasp (collected far from both natal and familiar colony collection sites). Each lure wasp was warmed to room temperature for several minutes after removal from the freezer, before recognition tests. During the bioassay, each lure was held with forceps and slowly introduced into the cage containing the experimental wasp on its natal nest. The lure was held about 1 cm from the nest and maintained for 1 min after the first contact between the experimental wasp and the lure (bite or simple antennal inspection). The three different lures were presented to experimental wasps in a random order and subsequent presentations were performed at least 30 min apart. Each lure wasp was used only once. The experimenter performing the lure presentations was blind to the origin of the lures and a second experimenter video recorded the behavioural tests. Videos were watched by a third observer, who was blind to the treatments used. The time spent by each experimental individual biting the lure wasp (Table S1) was counted for statistical analysis.

### Statistical Analyses

Duration of aggression of the experimental wasps towards the different lures (nestmates, alien, familiar) was analyzed with a non-parametric Friedman test. Post hoc tests (Wilcoxon signed-ranks tests) were used to assess whether a significant difference existed between pairs of treatments with a P value lower than α/number of comparisons (0.05/3 = 0.0167) considered significant. To test for any possible interaction between treatment and colony of origin on the wasp’s aggressive response, data were analyzed with a generalized linear model (GLZ) with Tweedie distribution, with the “number of aggressive acts” as dependent variable and “treatment” and “colony” as fixed factors. The GLZ revealed that there was no significant treatment x colony interaction term (Wald Chi-square = 53.795, *df* = 37, P = 0.482). For all statistical analyses we used SPSS 20.00 for Windows (SPSS Inc., Chicago, IL, U.S.A.).

## Results

**Figure 1:**
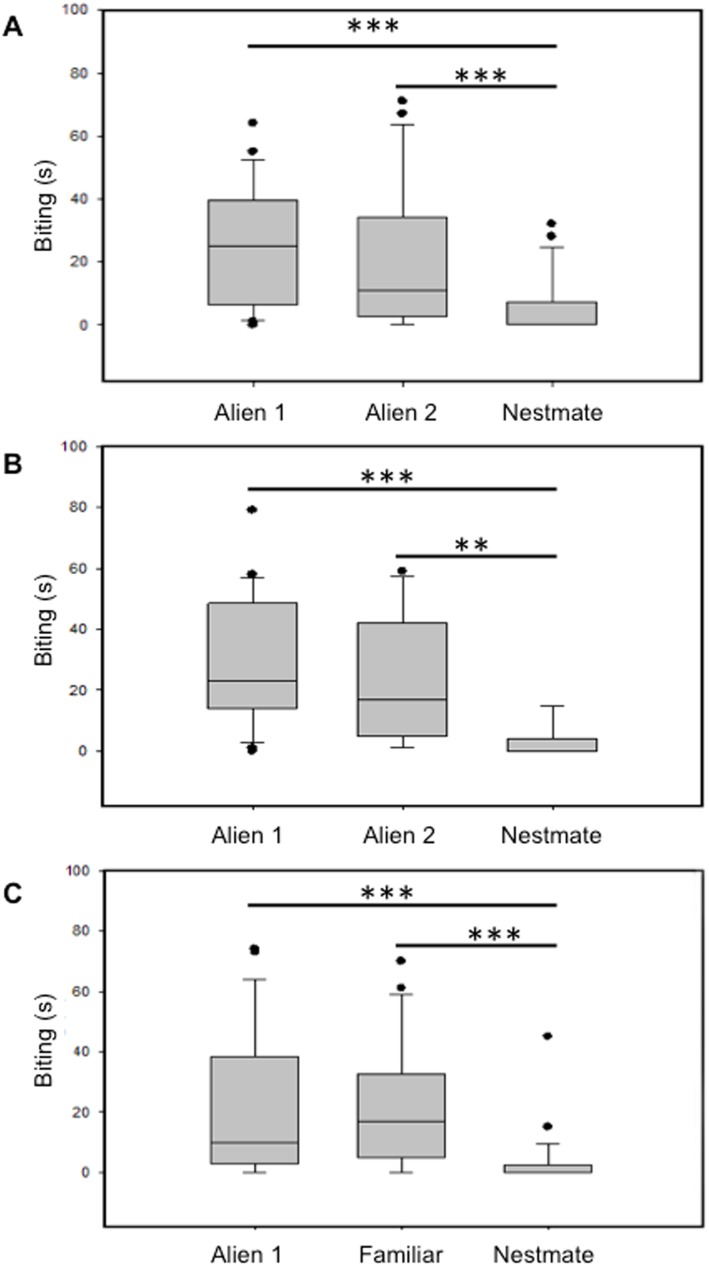
Behavioural tests. Aggressive responses (time spent biting) of experimental wasps towards lure wasps belonging to three different categories (nestmates, familiar, alien) for the three experimental conditions: A) “Control”: pre-eclosing workers were exposed for four days to the paper of their natal nest; B) “Neutral”: pre-eclosing workers were exposed for four days to no odours (filter paper); C) “Familiarized”: pre-eclosing workers were exposed for four days to the paper of a foreign nest. Thick horizontal lines represent medians, boxes are upper and lower quartiles and whiskers indicate the highest and lowest values excluding outliers (circles). **P<0.01; ***P<0.001.

We found significant differences in the time spent by the experimental wasps in biting the three categories of lure wasps in each of the three treatments (“Control”: χ^2^ = 17.761, N = 24, P<0.001; “Neutral”: χ^2^ = 35.293, N = 25, P<0.0001; “Familiarized”: χ^2^ = 28.645, N = 26, P<0.0001). In particular, “C” wasps spent more time biting alien than nestmate lures (nestmate vs alien 1: Z = 3.619, N = 24, P<0.001; nestmate vs alien 2: Z = 3.128, N = 24, P = 0.0017) but they made no differences between alien lures (Z = 0.8, N = 24, P = 0.424) (Figure 1A). Similarly, “N” wasps spent more time biting alien lures than nestmate lures (nestmate vs alien 1: Z = 4.286, N = 25, P<0.0001; nestmate vs alien 2: Z = 4.049, N = 25, P<0.0001), with no differences between alien lures (Z = 1.628, N = 25, P = 0.103) (Figure 1B). Finally, “F” wasps were equally aggressive towards alien and familiar lures (Z = 0.4, N = 26, P = 0.689), while they were significantly less aggressive towards nestmate lures (nestmate vs alien: Z = 3.733, N = 26, P = 0.0002; nestmate vs familiar: Z = 4.107, N = 26, P<0.0001) (Figure 1C). Therefore, the pattern of the wasps’ response was similar in the three experimental conditions, indicating no detectable effects of early olfactory experience through contact with the nest material on wasps’ recognition ability.

## Discussion and Conclusions

Our results show that in *P. dominula* the nest material is not the primary and fundamental source of recognition cues for the template formation during the first hours after emergence, as suggested by studies on other *Polistes* species [20]. Experimental workers of *P. dominula*, taken from their natal combs when the natural emergence occurs, are able to develop correct discrimination abilities regardless of their olfactory experience during the first four days of adult life. Neither the presence of alien nest fragments nor the total absence of nest material altered the wasps’ recognition abilities. This is a completely novel result concerning the ontogeny of nestmate recognition in *Polistes* wasps.

One possible explanation for our results is that *P. dominula* wasps may form the referent template at the adult stage from a direct contact with nestmates, as in some species of ants in which the template formation appears to be based on cues learned from other workers [40], [41], [42], [43]. During the first days of adult life, *P. dominula* wasps do not leave the nest and therefore the contact with nestmates is frequent. However, in our experimental procedure, wasps were isolated from other individuals for the first four days of adult life, and they spent only one day on their natal nest along with their nestmates before bioassays were performed. They may have had the opportunity to learn from their nestmates at that time. If this is the case, the sensitive period for learning is not strict in terms of time-window, as previously thought, but it might be context-dependent: the wasps could learn from nestmates once met. In order to investigate the possible importance of direct contact with conspecific individuals, it will be necessary to perform further experiments in which wasps are exposed only to conspecifics (nestmates or non-nestmates) during the earlier stages of adult life.

Nestmate recognition ability represents a common feature of all the species of *Polistes* investigated so far [11]. Nonetheless, the ontogeny of the mechanism underlying such discrimination ability has been experimentally tested only in the three American species (*P. fuscatus, P. carolina, P. metricus*; respectively [28], [29], [30]), out of more than 200 species belonging to the genus [32], and then generalized for all *Polistes* species, without direct experimental evidence. Different *Polistes* species, however, experience different colony structures and different ecological pressures that could somehow affect nestmate recognition mechanisms. Indeed, Queller and co-workers [38] found that, differently from American species (*P. bellicosus,* [44]; *P. fuscatus,* [45]; *P. carolina,* [46]), in an Italian population of *P. dominula*, 35% of nestmate foundresses in spring colonies are unrelated (a result later confirmed by Zanette and Field [47] for a Spanish population). Unrelated co-foundresses are unexpected as social insect colonies are usually composed by close relatives, so that helping behaviour can be favored by kin-selection [8], however, a shift of power can occur if an unrelated co-foundress usurps the colony from the previous queen [37], [48]. Furthermore, *P. dominula* represents the host species of two out of the three species of obligate interspecific social parasites known in the *Polistes* genus [49]. Thus, after either intra- or interspecific colony usurpation, the social structure a *P. dominula* colony can be dramatically altered with important consequences for the recognition system [50].

In particular, *P. dominula* wasps differ from the traditional ontogenetic model proposed for *Polistes* in two main aspects: nest material could not be the source of recognition cues for the template formation and/or the first hours after the emergence may not represent the sensitive period crucial for template acquisition. Alternative explanations for these differences may be plausible. One possibility is that, in *P. dominula* wasps, the first hours of adult life are not as sensitive as previously thought [20], but the nest material could still represent an important source of recognition cues to form the referent template later in life. There is strong evidence that the exposure to nest material is an essential step in the colony odour learning process, and thus in the template formation, in other social insects such as honeybees [51], [52], [53]. Moreover, the paper of *Polistes* wasps’ combs is a very good absorbent substrate rich of chemicals [21], [22], [23], [24]. The nest material is also the substrate marked by the dominant foundress with her own odour [54], [55], [56], and both intra- and inter-specific *Polistes* social parasites, soon after host nest invasion, perform an accurate abdominal stroking behaviour on nest surface, presumably to acquire the colony chemical profile and/or incorporate their own cues on the host nest [49], [57], [58], [59]. In this way, social parasites alter the source of recognition cues and the colonial reference template of the host to maximize their chances to be accepted in the usurped colony [50], [60], [61].

Given the importance of the nest as source of recognition cues for the learning process, we could also argue that wasps might modulate the beginning and the end of the sensitive period for learning on the basis of the presence of a relevant stimulus. Wasps could evaluate the stimuli present in their environment after birth and start the learning process only when meaningful stimuli appear. In our experiment, wasps were exposed during the first days of adult life to fragments of nest paper without brood or other adults. Then, all the individuals were returned to their natal nests for a day before performing the recognition bioassays, i.e., they were exposed to a novel and, possibly, more biologically relevant stimulus (a nest with alive brood and wasps). Empty nest fragments may not represent meaningful stimuli sufficient to create a recognition template. Consequently, the time-window for learning the recognition cues might have been extended and experimental wasps might have formed their “correct” templates on the second stimulus (i.e., their natal nests), due to a reversible imprinting-like phenomenon [62]. This would explain why wasps under all our experimental conditions recognized their nestmates. Alternatively, wasps could have learned the relevant cues for recognition first on the nest fragments provided during the exposure treatment, and then, updated the information once reintroduced on their own nests. Thus, the first template could have been replaced by a new one acquired through the exposure to a more relevant stimulus i.e. a nest with brood and/or adults. In order to investigate the possibility of an “imprinting reversibility” phenomenon it would be interesting to manipulate the wasps’ early experience to understand what is the most “relevant” stimulus for them.

Another possible explanation for our intriguing results might be occurrence of self-referent phenotype matching [14], [63], in which an individual learns the referent template from cues present on its own body. There are not many examples of self-referencing in social insects, but few studies carried out on honeybees have suggested the existence of this mechanism in the ontogeny of recognition abilities [64], [65]. Own cues might be suitable for learning, provided that they are available. Newly eclosed social insects, however, are reported to bear only little amounts of CHCs [17], [39], [66], [67], and we currently do not know whether CHCs of young individuals are over the perception threshold [68], [69], [70]. Moreover, *P. fuscatus* and *P. dominula* young wasps change significantly their CHCs mixture between 24 and 72 hours from eclosion, particularly with respect to abundance, relative abundance, and colony specificity of compounds [39], [71]. In our study, *P. dominula* workers were tested on the fifth day after emergence (four days in Petri dish and one day on their natal nests); therefore, it is likely that their CHCs profile were sufficiently developed to allow the template formation through a self-referencing process. However, newly emerged *P. dominula* workers (younger than 24 h) passively and readily (2 h of treatment) acquire chemical compounds onto the cuticle [39]. Therefore, the cuticle of our experimental workers could have acquired different chemical profiles accordingly to the treatments. In this case, we would expect different responses to recognition tests but we had similar results in all our bioassays. In particular, the treatment without nest odour suggests that self-referencing may occur. It is possible that newly emerged workers learn their own cues (genetic components) before acquiring colonial cues from the comb (environmental components) or they perform a sort of selective learning, i.e. by preferring the genetic component. Self-referent phenotype matching for template formation could be favored in *P. dominula* when there is a high risk to learn cues from unrelated nestmates. It has been suggested that the colony kin structure likely affects colonial odours [12] and *P. dominula* shows a strong relationship between CHCs composition and level of relatedness [72]. Therefore, in spring associations among unrelated individuals the compounds spread on the nest material could mirror the odour of several different unrelated individuals that share the nest. In this case, for a worker, the nest paper would not be a reliable source of recognition cues for template acquisition because it does not exactly correspond to the mother’s profile. A better reference source for template formation could be the own genetic cues through a self-referent matching process, which would allow nepotistic or selfish behaviour in generally heterogeneous colonies. Colony-level costs could preclude workers from replacing subordinate unrelated foundresses at an early stage of colony growth, but workers may delay their response and expel subordinates later in the season, thus seeking direct fitness benefits by producing males [73]. Also, self-referent phenotype matching would allow better recognition of social parasites. Indeed, *P. dominula* workers parasitized by *P. sulcifer*, after six weeks from the usurpation of their colony by the social parasite, show more developed ovaries and lay more eggs than non-parasitized *P. dominula* workers [74]. The development of workers’ ovaries could be due to an incomplete physiological control by the social parasite but also to the fact that workers can detect, through a basic nestmate recognition process, that their colony is being parasitized [74].

Finally, another possible explanation for our results is that other sensitive periods of a wasp’s life, such as pre-imaginal larval or pupal stages, could be important for the formation of the referent template in *P. dominula*. Wasp larvae and pupae are reared inside the nest cells and they are in close contact with the nest material for the entire duration of their developmental period. Ecological pressures such as social parasitism might have led to the evolution of an advantageous precocious cues learning. The opportunity to learn the referent template from the nest material before emergence could ensure that wasps are less “corruptible” to the parasites’ odour manipulation. On the other side, parasites normally usurp the host nests during the pre-workers phase [49] i.e., when workers are not eclosed yet. This could be interpreted as the result of an arm race in which parasites try to alter the referent template of the host during the pre-imaginal phase to ensure the collaboration of the first generation of host workers, which are crucial for the success of the parasite [49]. The ability to learn recognition cues during pre-imaginal stages have been recently proposed in a study focusing on recognition performed by *P. dominula* workers emerged in nests usurped by the facultative social parasite *P. nimphus* [75]. Moreover, a rather neglected phenomenon as pre-imaginal learning has been recently demonstrated to play a role in nestmate recognition in *Aphaenogaster senilis* ant [76], and thus it could be more widespread among social insects than previously thought.

Our study shows for the first time that the general mechanisms of recognition proposed for the paper wasps of the *Polistes* genus [20], which is actually very strict concerning the timing of template formation, is not applicable to all species within this genus and cannot be generalized. Although increasing progress on the study of the ontogeny of nestmate recognition abilities has been achieved and different underlying mechanisms have been proposed, future studies are needed to enlighten neglected phenomena such as pre-imaginal learning, self-referencing or reversible imprinting-like phenomena.

## Supporting Information

Table S1 Aggressive response (Time spent biting the lure wasp).
(XLSX)

## References

[1] Lorenz K (1937) The companion in the bird’s world. Auk. 54: 245–273.

[2] Michel GF, Tyler AN (2005) Critical period: A history of the transition from questions of when, to what, to how. Dev Psychobiol 46(3): 156–162. 15772973 10.1002/dev.20058

[3] Ramsay AO, Hess EH (1954) A laboratory approach to the study of imprinting. Wilson Bulletin 66: 196–206.

[4] Bolhuis JJ, Honey RC (1998) Imprinting, learning and development: From behaviour to brain and back. Trends Neurosci 21: 306–311. 9683323 10.1016/s0166-2236(98)01258-2

[5] Hinde RA (1961) The establishment of the parent-offspring relationship in birds with some mammalian analogies. In Thorpe W. and Zangwill O. (eds) Current Problems in Animal Behaviour. Cambridge University Press, Cambridge. 175–193.

[6] Knudsen EI (2004) Sensitive periods in the development of the brain and behavior. J Cognitive Neurosci 16(8): 1412–1425. 10.1162/0898929042304796 15509387

[7] Champalbert A, Lachaud JP (1990) Existence of a sensitive period during the ontogenesis of social behaviour in a primitive ant. Anim Behav 39(5): 850–859.

[8] Hamilton WD (1964) The genetical evolution of social behaviour I. J Theor Biol. 7: 1–16. 10.1016/0022-5193(64)90038-4 5875341

[9] Howard RW, Blomquist GJ (2005) Ecological, behavioral, and biochemical aspects of insect hydrocarbons. Annu Rev Entomol 50: 371–93. 15355247 10.1146/annurev.ento.50.071803.130359

[10] Dani FR (2006) Cuticular lipids as semiochemicals in paper wasps and other social insects. Ann Zool Fennici 43: 500–514.

[11] Bruschini C, Cervo R, Turillazzi S (2010) Pheromones in Social Wasps. In Vitamins and Hormones Gerald Litwack, editor. Burlington: Academic Press 83: 447–492. 10.1016/S0083-6729(10)83019-5 20831958

[12] Van Zweden JS, d’Ettorre P (2010) Nestmate recognition in social insects and the role of hydrocarbons. In: Insect Hydrocarbons: Biology, Biochemistry and Chemical Ecology, GJ Blomquist, AG Bagnères. Eds. Cambridge University Press, Cambridge 222–243.

[13] Crozier RH, Pamilo P (1996) Evolution of social insect colonies. Oxford University Press, Oxford. Pp. 306.

[14] d’Ettorre P, Lenoir A (2010) Nestmate recognition. In: Lach L, Parr CL, Abbott KL, eds. Ant ecology. Oxford: Oxford University Press. 194–209.

[15] Gamboa GJ, Reeve HK, Pfennig DW (1986a) The evolution and ontogeny of nestmate recognition in social wasps. Ann Rev Entomol 31(1): 431–454.

[16] Caubet Y, Jaisson P, Lenoir A (1992) Preimaginal induction of adult behaviour in insects. Q J Exp Psychol -B 44(3–4): 165–178.

[17] Lenoir A, Fresneau D, Errard C, Hefetz A (1999) “Individuality and colonial identity in ants: the emergence of the social representation concept” in Information Processing in Social Insects, eds C Detrain, J-L Deneubourg, JM Pasteels (Basel: Birkhäuser Verlag). 219–237.

[18] Bos N, d’Ettorre P (2012) Recognition of social identity in ants. Front Psychol 3. 10.3389/fpsyg.2012.00083 PMC3309994 22461777

[19] Gamboa GJ (1996) Kin recognition in social wasps. In “Natural History and Evolution of Paper Wasps,” (S Turillazzi and MJ West-Eberhard, Eds.). Oxford University Press, Oxford. 161–177.

[20] Gamboa GJ (2004) Kin recognition in eusocial wasps. Ann Zool Fennici 41: 789–808.

[21] Espelie KE, Hermann HR (1990) Surface lipid of the social wasp *Polistes annularis* (L.) and its nest and nest pedicel. J Chem Ecol 16: 1841–1852. 24263988 10.1007/BF01020498

[22] Espelie KE, Wenzel JW, Chang G (1990) Surface lipids of social wasp *Polistes metricus* say and its nest and pedicel and their relation to nestmate recognition. J Chem Ecol 16: 2229–2241. 24264089 10.1007/BF01026933

[23] Lorenzi MC (1992) Epicuticular hydrocarbons of *Polistes biglumis bimaculatus* (Hymenoptera, Vespidae): Preliminary results. Ethol Ecol Evol 3: 61–63 (Special Issue)..

[24] Singer TL, Camann MA, Espelie KE (1992) Discriminant analysis of cuticular hydrocarbons of social wasp *Polistes exclamans* Viereck and surface hydrocarbons of its nest paper and pedicel. J Chem Ecol 18: 785–797. 24253971 10.1007/BF00994615

[25] Cotoneschi C, Dani FR, Cervo R, Sledge MF, Turillazzi S (2007) *Polistes dominulus* (Hymenoptera: Vespidae) larvae have their own chemical signatures. J Insect Physiol 53: 954–963. 17498732 10.1016/j.jinsphys.2006.12.016

[26] Shellmann JS, Gamboa GJ (1982) Nestmate discrimination in social wasps: The role of exposure to nest and nestmates (*Polistes fuscatus*, Hymenoptera, Vespidae). Anim Behav 33: 331–332.

[27] Gamboa GJ, Reeve HK, Ferguson I, Wacker TL (1986) Nestmate recognition in social wasps: the origin and acquisition of recognition odours. Anim Behav 34: 685–695.

[28] Pfenning DW, Reeve HK, Shellmann JS (1983) Learned component of nestmate discrimination in workers of a social wasp, *Polistes fuscatus* (Hymenoptera, Vespidae). Anim Behav 31: 412–416.

[29] Pfenning DW, Gamboa GJ, Reeve HK, Shellmann JS, Reeve JS, et al (1983) The mechanism of nestmate discrimination in social wasps (Hymenoptera: Vespidae). Behav Ecol Sociobiol 13: 299–305.

[30] Singer TL, Espelie KE (1992) Social wasps use nest paper hydrocarbons for nestmate recognition. Anim Behav 44(1): 63–68.

[31] Dreier S, d’Ettorre P (2009) Social context predicts recognition systems in ant queens. J Evol Biol 22(3): 644–649. 19170823 10.1111/j.1420-9101.2008.01668.x

[32] Carpenter JM (1996) Phylogeny and biogeography. In “Natural History and Evolution of Paper Wasps” (S Turillazzi and MJ West-Eberhard, Eds.). Oxford University Press, Oxford. 18–57.

[33] Pardi L (1996) “Polistes: analysis of a society.” Natural history and evolution of paper wasps Ed. by S Turillazzi & MJ West- Eberhard. Oxford: Oxford University Press. 1–17.

[34] Cervo R, Zacchi F, Turillazzi S (2000) *Polistes dominulus* (Hymenoptera, Vespidae) invading North America: some hypotheses for its rapid spread. Insect Soc 47(2): 155–157.

[35] Liebert AE, Gamboa GJ, Stamp NE, Curtis TR, Monnet KM, et al. (2006) Genetics, behavior and ecology of a paper wasp invasion: *Polistes dominulus* in North America. In Ann Zool Fenn 43 (5–6) p. 595. Helsinki: Suomen Biologian Seura Vanamo, 1964-.

[36] Pardi L (1942) Ricerche sui Polistini. V. La poliginia iniziale in *Polistes gallicus* (L.). Boll Ist Entomol Univ Bologna 14: 1–106.

[37] Pardi L (1946) Ricerche sui Polistini VII. La “dominazione” ed il ciclo ovarico annuale di *Polistes gallicus* (L.). Boll Ist Entomol Univ Bologna 15: 25–84.

[38] Queller DC, Zacchi F, Cervo R, Turillazzi S, Henshaw MT, et al (2000) Unrelated helpers in a social insect. Nature 405(6788): 784–787. 10866197 10.1038/35015552

[39] Lorenzi MC, Sledge MF, Laiolo P, Sturlini E, Turillazzi S 2004 Cuticular hydrocarbon dynamics in young adult *Polistes dominulus* (Hymenoptera: Vespidae) and the role of linear hydrocarbons in nestmate recognition systems. J Insect Physiol 50: 935–941. 15518661 10.1016/j.jinsphys.2004.07.005

[40] Morel L (1983) Relation entre comportement agressif et privation sociale précoce chez les jeunes fourmis immatures de la fourmi *Camponotus vagus* Scop. (Hymenoptera: Formicidae). C R Hebd Séances. Acad Sci Série D 296: 449–452.

[41] Morel L (1988) Ontogènese de la reconnaissance des membres de la société chez *Camponotus floridanus* (Hymenoptera: Formicidae). Role de l’expérience sociale précoce. Biologie du Comportement 13: 59–72.

[42] Boulay R, Lenoir A (2001) Social isolation of mature workers affects nestmate recognition in the ant *Camponotus fellah* . Behav Process 55: 67–73. 10.1016/s0376-6357(01)00163-2 11470498

[43] Boulay R, Katzav-Gozansky T, Vander Meer RK, Hefetz A (2003) Colony insularity through queen control on worker social motivation in ants. Proc R Soc Lond B 270: 971–977. 10.1098/rspb.2002.2325 PMC1691331 12803913

[44] Field J, Solis CR, Queller DC, Strassmann JE (1998) Social and genetic structure of paper wasp cofoundress associations: tests of reproductive skew models. Am Nat 151: 545–563. 18811376 10.1086/286140

[45] Reeve HK, Starks PT, Peters JM, Nonacs P (2000) Genetic support for the evolutionary theory of reproductive transactions in social wasps. Proc R Soc Lond B 267: 75–79. 10.1098/rspb.2000.0969 PMC1690491 10670956

[46] Seppa P, Queller DC, Strassmann JE (2002) Reproduction in foundress associations of the social wasp, *Polistes carolina*: conventions, competition, and skew. Behav Ecol 13: 531–542.

[47] Zanette LR, Field J (2008) Genetic relatedness in early associations of *Polistes dominulus*: from related to unrelated helpers. Mol Ecol 17(11): 2590–2597. 18482265 10.1111/j.1365-294X.2008.03785.x

[48] Leadbeater E, Carruthers JM, Green JP, Rosser NS, Field J (2011) Nest inheritance is the missing source of direct fitness in a primitively eusocial insect. Science 333(6044): 874–876. 21836014 10.1126/science.1205140

[49] Cervo R (2006) *Polistes* wasps and their social parasites: an overview. Ann Zool Fenn 43: 550–563.

[50] Lorenzi MC (2006) The result of an arms race: the chemical strategies of Polistes social parasites. In Ann Zool Fenn 43(5–6) 550–563. Helsinki: Suomen Biologian Seura Vanamo, 1964-.

[51] Breed MD, Garry MF, Pearce AN, Bjostad L, Hibbard B, et al (1995) The role of wax comb in honeybee nestmate recognition: genetic effects on comb discrimination, acquisition of comb cues by bees, and passage of cues among individuals. Anim Behav 50: 489–496.

[52] Breed MD, Leger EA, Pearce AN, Wang YJ (1998) Comb wax effects on the ontogeny of honey bee nestmate recognition. Anim Behav 55: 13–20. 9480667 10.1006/anbe.1997.0581

[53] D’Ettorre P, Wenseleers T, Dawson J, Hutchinson S, Boswell T, et al (2006) Wax combs mediate nestmate recognition by guard honey bees. Anim Behav 71: 773–779.

[54] Dani FR, Cervo R, Turillazzi S (1992) Abdomen stroking behaviour and its possible functions in *Polistes dominulus* (Christ) (Hymenoptera Vespidae). Behav Process 28: 51–58. 10.1016/0376-6357(92)90048-I 24924790

[55] Van Hooser CA, Gamboa GJ, Fishwild TG (2002) The function of abdominal stroking in the paper wasp, *Polistes fuscatus* (Hymenoptera Vespidae). Ethol Ecol Evol 14(2): 141–148.

[56] Dapporto L, Santini A, Dani FR, Turillazzi S (2007) Workers of a *Polistes* paper wasp detect the presence of their queen by chemical cues. Chem Senses 32(8): 795–802. 17644826 10.1093/chemse/bjm047

[57] Cervo R, Turillazzi S (1989) Nest exchange experiments in *Polistes gallicus* (L.) (Hymenoptera Vespidae). Ethol Ecol Evol 1(2): 185–193.

[58] Cervo R, Lorenzi MC (1996) Behaviour in usurpers and late joiners of *Polistes biglumis bimaculatus* (Hymenoptera, Vespidae). Insect Soc 41: 1–11.

[59] Zacchi F, Cervo R, Turillazzi S (1996) How *Polistes semenowi*, obligate social parasite, invades the nest of its host, *Polistes dominulus* (Hymenoptera, Vespidae). Insect Soc Life 1: 125–130.

[60] Lorenzi MC, Cometto I, Marchisio G (1999) Species and colony components in the recognition odor of young social wasps: their expression and learning (*Polistes biglumis* and *P. atrimandibularis*; Hymenoptera: Vespidae). J Insect Behav 12(2): 147–158.

[61] Turillazzi S, Sledge MF, Dani FR, Cervo R, Massolo A, et al (2000) Social hackers: integration in the host chemical recognition system by a paper wasp social parasite. Naturwissenschaften 87(4): 172–176. 10840803 10.1007/s001140050697

[62] Bolhuis JJ (1991) Mechanisms of avian imprinting: a review. Biol Rev 66(4): 303–345. 1801945 10.1111/j.1469-185x.1991.tb01145.x

[63] Mateo JM (2004) Recognition systems and biological organization: the perception component of recognition. Ann Zool Fenn 41: 729–745.

[64] Getz WM, Smith KB (1983) Genetic kin recognition: honey bees discriminate between full and half sisters. Nature 302: 147–148.

[65] Getz WM, Smith KB (1986) Honey bee kin recognition: learning self and nestmate phenotypes. Anim Behav 34, 1617–1626.

[66] Stuart RJ (1992) Nestmate recognition and the ontogeny of acceptability in the ant, *Leptothorax curvispinosus.* . Behav Ecol Sociobiol 30: 403–08.

[67] Breed MD, Perry S, Bjostad LB (2004) Testing the blank slate hypothesis: why honey bee colonies accept young bees. Insect Soc 51: 12–16.

[68] Cini A, Gioli L, Cervo R (2009) A quantitative threshold for nestmate recognition in a paper social wasp. Biol Lett 5: 459–461. 19411275 10.1098/rsbl.2009.0140 PMC2781916

[69] Ichinose K, Lenoir A (2010) Hydrocarbons detection levels in ants. Insect Soc 57: 453–455.

[70] Cappa F, Bruschini C, Cipollini M, Pieraccini G, Cervo R (2014) Sensing the intruder: a quantitative threshold for recognition cues perception in honeybees. Naturwissenschften 1–4. 10.1007/s00114-013-1135-1 24402686

[71] Panek LM, Gamboa GJ, Espelie KE (2001) The Effect of a Wasp’s Age on Its Cuticular Hydrocarbon Profile and Its Tolerance by Nestmate and Non-Nestmate Conspecifics (*Polistes fuscatus*, Hymenoptera: Vespidae). Ethology 107: 55–63.

[72] Dani FR, Foster KR, Zacchi F, Seppa P, Massolo A, et al (2004) Can cuticular lipids provide sufficient information for within-colony nepotism in wasps? Proc R Soc Lond B 271(1540): 745–753. 10.1098/rspb.2003.2646 PMC1691643 15209109

[73] Monnin T, Cini A, Lecat V, Fédérici P, Doums C (2009) No actual conflict over colony inheritance despite high potential conflict in the social wasp *Polistes dominulus* . Proc R Soc Lond B 276(1662): 1593–1601. 10.1098/rspb.2008.1739 PMC2660987 19203923

[74] Cini A, Nieri R, Dapporto L, Monnin T, Cervo R (2013) Almost royal: incomplete suppression of host worker ovarian development by a social parasite wasp. Behav Ecol Sociobiol 1–9.

[75] Costanzi E, Bagnères AG, Lorenzi MC (2013) Changes in the Hydrocarbon Proportions of Colony Odor and Their Consequences on Nestmate Recognition in Social Wasps. PLoS ONE 8(5): e65107. 23734237 10.1371/journal.pone.0065107 PMC3667189

[76] Signorotti L, Jaisson P, d’Ettorre P (2014) Larval memory affects adult nestmate recognition in the ant *Aphaenogaster senilis* . Proc R Soc B 281: 20132579. 10.1098/rspb.2013.2579 PMC3843841 24258719

